# How Women with Endometriosis Experience Health Care Encounters

**DOI:** 10.1089/whr.2020.0099

**Published:** 2020-12-07

**Authors:** Agneta Pettersson, Carina M. Berterö

**Affiliations:** ^1^ Swedish Agency for Health Technology Assessment and Assessment of Social Services (SBU), Stockholm, Sweden.; ^2^ Division of Nursing Science and Reproductive Health, Department of Health, Medicine and Caring Sciences, Faculty of Medicine, Linköping University, Linköping, Sweden.

**Keywords:** endometriosis, health care encounter, hermeneutics, meta-synthesis, qualitative research, women's experiences

## Abstract

***Objective:*** The aim of this meta-synthesis was to synthesize and interpret the available qualitative studies to increase our understanding and extend knowledge about how women with endometriosis experience health care encounters.

***Methods:*** The literature review was carried out using CINAHL, Psychinfo, Academic Search Premier, PubMed, and Scopus, from 2000 to 2018, and was limited to articles in English. Articles were only included if they reported original relevant research on endometriosis and women experiences.

***Results:*** The meta-synthesis was based on 14 relevant studies. They included 370 women with diagnosed endometriosis, 16–78 years of age. Three fusions were identified and interpreted in this meta-synthesis. The first was: Insufficiency knowledge, where the physicians could judge the symptoms to be normal menstruation without examining whether there were other underlying causes. The second fusion was Trivializing—just a women's issue, where the physicians thought that the symptoms were part of being a woman, and women's' discomfort was trivialized or completely disregarded. The third fusion was Competency promotes health, where the insufficiency of knowledge became a minor concern if women had a supportive relationship with their physician and the physician showed interest in their problems.

***Conclusions:*** Women with endometriosis experience that they are treated with ignorance regarding endometriosis in nonspecialized care. They experience delays in both their diagnosis and treatment and feel that health care professionals do not take their problems seriously. In addition, it appears that increased expertise and improved attitudes among health care professionals could improve the life situation of women with endometriosis.

## Introduction

Endometriosis is a chronic, inflammatory, and estrogen-dependent gynecological disease associated with pain and infertility that affects women of reproductive age.^1^ Endometriosis has been here throughout the ages and strikes women worldwide.^2^ It is estimated that 176 million women worldwide may be living with endometriosis. Approximately 10% of women of reproductive age are affected.^3^ Endometriosis occurs when endometrial cells, which normally line the uterus, grow outside the uterus. This tissue implants in, and forms lesions on, other organs, including the ovaries, bowel, bladder, and the Pouch of Douglas.^4^

Gynecological symptoms of endometriosis include, dysmenorrhea, dyspareunia, chronic pelvic pain, bleeding, fatigue, and, in some cases, urological or gastrointestinal symptoms (such as dysuria, dyschezia). Compromised fertility or infertility are other symptoms associated with endometriosis.^5,6^

Laparoscopy is the most common procedure used to diagnose endometriosis, ideally confirmed by histology.^7,8^ Delay in the diagnosis of endometriosis may have several causes such as false diagnosis and normalization of symptoms,^9^ but delay often occur because the gold standard for disease confirmation is using laparoscopy and histology. Such delays can adversely affect reproductive potential and functional outcomes.^8^ Endometriosis affects a woman's life considerably. A recent systematic review,^10^ found only 18 qualitative articles on endometriosis. This review showed that endometriosis affected all areas of a woman's life; sex life, social life, and work life, and medical experiences, symptom, and infertility are in some way intertwined and added distress to life.^10^ This review also pointed out that there were few studies on women's experiences of endometriosis-associated infertility and of the impact of reduced social participation on perceived support and emotional wellbeing. There are also few systematic reviews or meta-analysis that address psychological care for women with endometriosis.^11,12^ These reviews focus on treatment or interventions that may be promising in reducing endometriosis pain, anxiety, depression, stress, and fatigue. Another area where there is little, or no research is how women with endometriosis experience health care encounters. Such knowledge is important for providing high-quality care. Therefore, the objective of this meta-synthesis was to synthesize and interpret the available qualitative studies to increase our understanding and extend knowledge about how women with endometriosis experience health care encounters.

## Materials and Methods

This article is an update of a qualitative meta-synthesis conducted by the Swedish Council for Health Technology Assessment as part of a report on the diagnosis and treatment of Endometriosis.^13^ This is a qualitative meta-synthesis, that is, an interpretive integration of qualitative findings that offers more than the sum of the individual data sets because it provides an innovative interpretation of the separate findings.^14–16^ The new findings and conclusions are derived from examining all the articles in a sample as a collective group, presenting interpretations that are representative since it is based on several articles.^14^ Qualitative meta-synthesis allows for a broader approach to evidence-based research and practice by expanding how knowledge can be generated and used in the researched area.^17,18^ A meta-synthesis is the outcome of a metadata method. It is a distinct approach to new inquiry based on critical interpretation of existing qualitative studies.^16^ “It creates a mechanism by which the nature of interpretation is exposed and the meanings that extend well below those presented in the available body of knowledge can be generated. As such, it offers a critical, historical and theoretical analytic approach to making sense of qualitative knowledge.”^16, p2^ The review/meta-synthesis was not registered in the international prospective register of systematic reviews (PROSPERO), since the data were already extracted, and the analysis was conducted.

### Sample

**FIG. 1.**
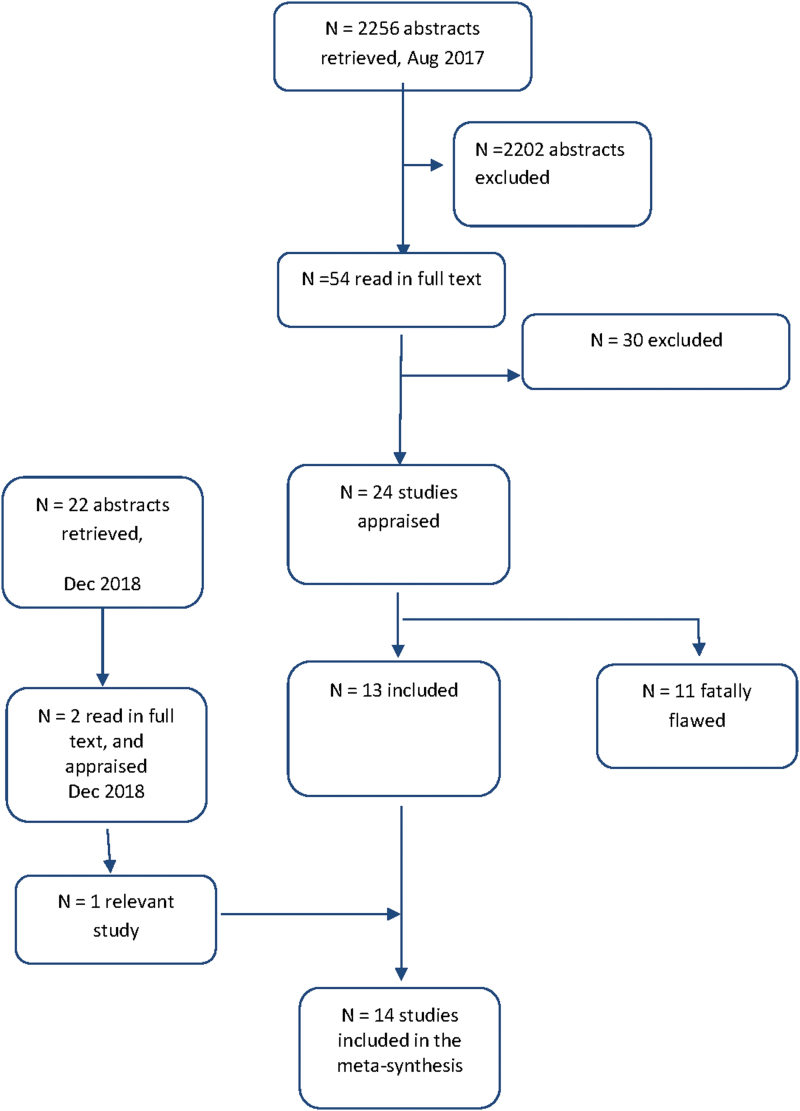
Flowchart of the literature search process.

Relevant qualitative research studies were retrieved on two occasions. A comprehensive search in the bibliographic databases CINAHL, Psych info, Academic Search Premier, PubMed, and Scopus was conducted on August 30, 2017. The articles had to meet the following inclusion criteria: (1) have a focus on women with endometriosis; (2) make explicit references to the use of qualitative research/studies or mixed methods, where the qualitative findings were reported separately; (3) have a focus on women's perspectives and experiences of living with endometriosis and how they experienced the encounters with health care; (4) published from 2000 to August 2017 and written in English. Exclusion criteria were quantitative research and literature reviews. An additional, selective, search in PubMed was conducted in December 2018, using the search terms *endometriosis*, *women's experiences*, and *health care encounters.* As shown in the flowchart in Figure 1, 24 studies were retrieved for consideration. A complementary search identical with 2018 was performed in December 2019. No more studies were retrieved.

### Meta-method

The meta-method requires an analysis of the rigor and soundness of the research methods used in each of the studies reviewed to determine the appropriateness of the methods.^15^ The meta-method procedure had two steps. First, the studies were evaluated, with an emphasis on research design and data collection methods, to ensure that the article met the study's inclusion criteria.^16^ Of the 54 articles, 30 were excluded. One of these evaluated a training program and used a survey with open-ended questions. This article was retained for validation of our results. Second, the studies were appraised; incorporating the reading guide for both an individual appraisal of each possible study considered for inclusion, together with comparative appraisal across studies. Key features of the studies were summarized, and a cross-study display was developed presenting the 14 articles selected for the study (Table 1). There is a large amount of data in each study, so the recommendation is that 10–12 studies is an ideal number for a meta-synthesis.^17^ The studies represented the following disciplines: sociology, medicine, nursing, and physiotherapy (Table 1).

**Table 1.** Showing primary research features in articles included in analysis

	Aim Underpinning theory	Setting Participants	Sampling	Data collection	Analysis	Measures to support trustworthyness
Ballard et al.^23^ United Kingdom	*Aim of study* To investigate the reasons women experience delays in diagnosis of endometriosis and the impact of this *Underpinning theory* Not described	*Setting* Hospital pelvic pain clinic *Participants* 32 women Age: 16-47 years; (median 32) Years with pelvic pain: median 15 years	*Sampling* Not described *Inclusion criteria* confirmed or suspected endometriosis	*Data collection* Semi structured, face-to-face interviews, most often conducted in the home of the interviewee; 60-120 minutes *Interviewer* The author, social scientist	*Analysis* Thematic analysis where experiences and beliefs that women expressed were interpreted for key themes. Only women with confirmed endometriosis were included in the analysis *Analysts* Initial analysis by the author (a social scientist), refined after discussions with a pelvic pain specialist (gyneacologist) and a social scientist	Development of the coding frame and the initial analysis was carried out by a social scientist. The findings were then discussed with a consultant gynecologist and specialist in pelvic pain, and a social scientist. Based on these discussions, the analysis was further refined
Denny & Mann^24^ United Kingdom	*Aims of study* Explore experiences from primary care. Reanalysis of data from Denny 2004 *Underpinning theory* ?	*Setting* A clinic for endometriosis at a specialist women's hospital *Participants* 30 women Age: 19 - 44 years, (mean 31) Diagnostic delay: mean 5,65 years (0-18 years)	*Sampling* Purposeful. *Inclusion criteria* Laparoscopically verified endometriosis	*Data collection* Semi structured interview based on a story-telling approach, in their home or at the clinic; 30-50 minutes Probing for primary care if not mentioned spontaneously *Interviewer* The author, a social scientist	*Analysis* Thematic analysis (Bryman) *Analysts* The two authors, one social scientist and one gynecologist	Respondent validation of the themes Although there was no methodological triangulation, rigour was achieved in analysis as both authors and the women who participated in the study agreed the analytical themes as relevant and arising from the data.
Denny^25^ United Kingdom	*Aim of study* Explore women's experience of living with endometriosis. One-year follow- up *Underpinning theory* Feminist approach	*Setting* A clinic for endometriosis at a specialist women's hospital *Participants* Interviews: 27 women; Age: 19 - 44 years, (mean 31) Diary: 19 of these women	*Sampling* Purposeful (interviews) Not reported (diaries)	*Data collection* Semi structured interview based on a story-telling approach, in their home or at the clinic; 30-50 minutes Diary on endometriosis for one menstrual cycle; completed by 7 women *Interviewer* The author, a social scientist	*Analysis* Narrative analysis *Analysts* Only one author, social scientist	Respondent validation of the themes the women who participated in the study agreed the analytical themes as relevant and arising from the data.
Facchin et al.^27^ Italy	*Aim of study* Provide a broader understanding on how endometriosis affects psychological health *Underpinning theory* Grounded theory	*Setting* Tertiary level referral center for treatment of endometriosis *Participants* 74 women Age: 24 -50 years	*Sampling* Theoretical sampling Consecutively recruited *Inclusion criteria* Self-referred for treatment, surgically verified diagnosis, different forms of endometriosis	*Data collection* Face- to face interviews with a story-telling approach, conducted at the hospital Time: average 45 minutes *Interviewer* Trained psychologists including the first author	*Analysis* Constant comparative (Corbin & Strauss) *Analysts* Three, working independently	All emergent themes were continuously discussed in the research team Findings were presented to expert gynecologists and female members of a non-for-profit endometriosis association Discrepancies were discussed until consensus was reached
Gilmour et al.^28^ New Zealand	*Aim of study* Explore the perceptions of living with endometriosis *Underpinning theory* Feminist research principles	*Setting* Local endometriosis support group *Participants* 18 women Age: 16 to 45 years Diagnostic delay: 5-10 years	*Sampling* Interested women from the support group contacted the researchers after information about the project	*Data collection* Unstructured, interactive interview *Interviewer* Not described, but familiar with endometriosis and knowledgeable how to handle emotional reactions during the interview	*Analysis* Thematic analysis *Analysts* The authors, with a nursing background and working as researchers at a department for health and social services	Continuous collaboration with the support group Emerging themes were presented at two meetings and verified by the participants
Grundström et al.^31^ Sweden	*Aim of study* Identify and describe the experiences of health care encounters for women with endometriosis *Underpinning theory* phenomenology	*Setting* A university and a central hospital clinic *Participants* 9 women consecutively invited by three gynecologists in charge of their endometriosis treatment Age: 23-55 years (median 37 )	*Sampling* Purposive sampling *Inclusion criteria* Age >18 years Laparoscopy-verified endometriosis	*Data collection* Semi-structured interviews in the home or a separate room at the hospital library Length: 33-113 min (median 64 min) *Interviewer* Midwife and Doctoral student	*Analysis* Moustaka's modification of the Stevick-Colaizzi-Keen method (adding interpretation) *Analysts* Three researchers (two with midwife background, one a PhD student and the other a researcher, the third with a nursing background and researcher) conducted the analysis independently followed by discussion and consensus about the essence.	The methods used to establish trustworthiness in this study were reporting the audit trail (i.e., describing every step of the data collection and analysis), and using quotations to illustrate the themes and to show that the findings were grounded in the women's stories. To avoid overinterpretation, the research team analysed the data separately, discussed the analysis and found agreement in the interpretation.
Hållstam et al.^32^ Sweden	*Aim of study* To examine women's experience of painful endometriosis including long-term aspects, social consequences, impact of treatment and development of own coping strategies *Underpinning theory* Grounded theory	*Setting* At the specialized pain clinic of a tertiary center *Participants* 13 women Age: 24–48 years (mean 36)	*Sampling* Purposive sampling *Inclusion criteria* follow-up study after treatment for chronic pain at the clinic twenty-nine women were identified as having endometriosis	*Data collection* Semi-structured interviews most in a secluded place at the hospital, three in patients' homes, one at a workplace and one in a public library Length; 43- 82 min (mean 59 min). *Interviewer* Female nurse Female physiotherapist	*Analysis* Grounded theory (Corbin & Strauss) *Analysts* Two researchers (one nurse and one physiotherapist with experience of pain treatment and endometriosis and rehabilitation)	To ensure credibility research triangulation was performed. A peer review was done involving a gynecologist, a midwife, an anesthesiologist, a pain specialist and a physiotherapist all with experience of patients with endometriosis
Huntington & Gilmour^29^ New Zealand	*Aim of study* To explore women's perceptions of living with endometriosis, its effects on their lives and the strategies used to manage their disease. *Underpinning theory* Feminist research principles	*Setting* Local endometriosis support group *Participants* 18 women Age: 16 to 45 years Diagnostic delay: 5-10 years	*Sampling* Information sheets about the project and consent forms were distributed via newsletter *Inclusion criteria*	*Data collection* Individual, semi-structured, audio taped, interactive interviews *Interviewer* Not described, but familiar with endometriosis and knowledgeable how to handle emotional reactions during the interview	*Analysis* Thematic analysis *Analysts* The authors, with a nursing background and working as researchers at a department for health and social services	All texts were read, compared and tentative themes identified. Validity or ‘trustworthiness’ of the data in qualitative research relates to how well the data represents the experiences of the participants To determine the validity of the data from this research the findings were presented orally at two meetings of the endometriosis support group
Markovic et al.^19^ Australia	*Aim of study* to enrich our understanding of the relationship between the patient's socio-demographic background and health-related phenomena, by identifying distinctive differences among women's narratives. *Underpinning theory* Grounded theory—but influenced by endurance	*Setting* Women residing in the state of Victoria (Australia), with various gynecological conditions *Participants* 30 women Age 20 to 78 years (mean 43.9)	*Sampling* Information about the study was disseminated through community newspapers and notice boards; snowball sampling also occurred. *Inclusion criteria* Women self-selected	*Data collection* In-depth interviews, lasting for about 60 minutes *Interviewer*	*Analysis* Grounded theory (Corbin & Strauss) *Analysts*	This was an iterative process in which all authors read the transcripts and developed the coding book. They first identified the themes within individual transcripts and then checked them across narratives. The themes were identified inductively, by careful reading of the interview data, but also by searching for themes identified in prior research in the area of women's reproductive health, as presented in the introduction. Themes were included in the grounded theory only if a significant number of women (about half) spoke about them
Moradi et al.^20^ Australia	*Aim of study* to explore women's experiences of endometriosis and its impact, involving three different age groups recruited either from both a hospital clinic and the community. *Underpinning theory*	*Setting* 23 women from a dedicated Endometriosis Centre at one public teaching hospital in Canberra and 12 women from the community (who had not attended the Centre) *Participants* 35 women Age 17 to 53 years (mean 31.1)	*Method* women was purposefully recruited *Inclusion criteria* confirmed diagnosis of endometriosis (via laparoscopy) for at least a year, who were able to understand and speak English, and had no other chronic disease.	*Data collection* Focus groups interviews lasting for about 2.5 h *Interviewer* Two experienced health professionals with practical knowledge about endometriosis and interviewing skills.	*Analysis* Thematic analysis (Braun & Clarke) *Analysts* The whole research team was involved?	Rigour refers to the quality of qualitative enquiry and is used as a way of evaluating qualitative research. Seven participants from different focus groups were asked to check a transcription of their responses and confirmed its accuracy.
Jones et al.^26^ United Kingdom	*Aim of study* Explore and describe the impact of endometriosis on quality of life *Underpinning theory* Grounded theory to generate categories and concepts	*Setting* Gynecology outpatient clinic *Participants* 24 women (until theoretical saturation) Age:21,5-44 years (mean 32,5)	*Method* Theoretical sampling to cover different disease stages and symptom profiles *Inclusion criteria* Laparoscopically verified endometriosis	*Data collection* Semi-structured, in depth interviews at the hospital Mean time: 55 min *Interviewer* the researcher had no personal experience of endometriosis and only very basic knowledge of its symptoms before the interviews started	*Analysis* Constant comparative method *Analysts* Not described	Independent coding for some transcripts by a research nurse To reduce interviewer bias and to check whether the codes adequately reflected the emerging areas of HRQoL, a research nurse also went through some of the transcripts. The same themes were identified and the interviewees' dialogues were interpreted in the same way.
Roomaney & Kagee^30^ South Africa	*Aim of study* To explore, understand and describe HRQOL among South African women diagnosed with endometriosis. *Underpinning theory* Quality of life	*Setting* In both the private and public health systems at the Western Cape Province of South Africa (gynaecological departments/practices) *Participants* 25 women laparoscopically diagnosed with endometriosis Age: 25- 42 years (average age 33)	*Method* Convenience sampling *Inclusion* be surgically diagnosed with endometriosis, be 18 years or older and have experienced symptoms during the 3 months prior to being interviewed	*Data collection* Semi-structured interviews at participants' homes, places of work, the researcher's office or coffee shops Length: 31- 84 minutes *Interviewer* Not described	*Analysis* Thematic analysis (Braun and Clarke) *Analysts* The two authors	Both authors checked and re-checked the codes to ensure consistency in the data analysis. In addition, an independent coder was employed to verify the data analysis. Five interviews were coded independently and then compared. Any differences between codes were discussed until a consensus was reached regarding the labelling of codes. A code-book was developed during this process, and the first author used the code-book to code. the remaining interviews. We reviewed samples of coding of the data in order to enhance trustworthiness of analysis.
Seear^21^ Australia	*Aim of study* Examine the potentially broader application of these findings for the study of menstrual pain and chronic pelvic pain conditions more generally *Underpinning theory* a ‘discrediting attribute’ (Goffman, 1963) the ‘menstrual etiquette’ (Laws, 1990)	*Setting* From a qualitative study conducted in Australia *Participants* 20 women Age:24 - 55 years (mean 34).	*Method* snowball sampling and advertisement was also placed in the newsletter of an Australian support group	*Methods* semi-structured interviews Length of interviews*:* 45min to 2 h *Interviewer* Not described	*Analysis* Secundary analysis- (Miles and Huberman) *Analysts* Not described	A system of diagrams or ‘charts’ were used to display the data and the relationships between emergent themes. Following this process, the researcher returned to the original transcripts of interviews several times to check that the themes and concepts that I had been developed were supported by the data. Any negative cases were noted.
Young et al.^22^ Australia	*Aim of study* Explore experiences of health care related to endometriosis and fertility *Underpinning theory* Not described	*Setting* Non-clinical *Participants* 26 women, the majority in their 30s	*Method* invitation by advertisements. After 20 interviews purposeful sampling was applied to ensure diversity *Inclusion criteria* At least 18 years Surgically verified endometriosis	*Methods* In depth, semi-structured interviews, face-to face or over the phone Mean time: 63 minutes *Interviewer* First author	*Analysis* Thematic analysis (Braun & Clarke) *Analysts* Initial analysis by the first author and then all authors participated in the analysis and interpretation of data,	Analysis, the hierarchy of themes, and final categories of data were discussed among all authors and results were decided by agreement.

HRQoL, health-related quality of life.

The size of the research sample reported in each article ranged from 9 to 74, with a total sample size of 370 (mean sample size 26). The women in these studies were 16–78 years of age and represented different countries: Australia,^18–21^ Great Britain,^22–25^ Italy,^26^ New Zealand,^27,28^ South Africa,^29^ and Sweden.^30,31^ The data from the 370 women were based on individual interviews, in one case combined with focus group interviews, and one study was based on focus group interviews. All articles had been published from 2004 to 2018. In the second step, the appraisal process contributed to an understanding of how the methodology used in the original research had been applied to study a phenomenon and how that methodology had shaped the researcher's knowledge. The research designs of the primary research articles were compared and contrasted to identify the underlying assumptions of research methodologies, as well as the findings reported.^13.15^ The authors of the articles meeting the criteria for inclusion in this study described the methods used as qualitative, that is, the studies used phenomenology, narrativism, thematic analysis, grounded theory, and constant comparative analysis. Some researchers used thematic analysis and narrativism for secondary analysis. Analysis revealed that there was a variation in the rigor with which the tenets of the identified method were applied, as well as a range in the quality and quantity of direct quotes available to the analysts of this meta-synthesis.

### Preparing the findings for meta-synthesis

The quality of the way in which findings were presented in the articles varied; most articles presented raw data as thematic surveys and/or direct quotations from participant interviews. The metadata analysis used the technique of hermeneutic appraisal^33^ to extract statements from the articles' findings to evaluate the horizon of the text. The analysts then interpreted these statements within the context of a guiding question: Is this about being perceived or about the health care encounter. Working inductively from these interpretations, the researchers were able to identify possible fusions.^15,16^

## Results

The meta-synthesis resulted in three fusions: Insufficient knowledge, Trivializing—just a women's issue, and Competence promotes health.

### Insufficient knowledge

The women often stated that they had repeated problems that the cause of the symptoms seemed to be unknown and they had not been diagnosed. The women felt that the physicians lacked knowledge of the disease, which led to the diagnosis being delayed and that they did not receive adequate treatment. The insufficient knowledge could be demonstrated in several ways. The physicians could judge the symptoms as normal menstruation without examining whether there were other underlying causes. The physicians were annoyed when the women pointed out that their symptoms were similar to those of other women with diagnosed endometriosis. In other cases, incorrect diagnoses were made. The handling of the women's case, both before and after diagnosis, was often based on medical myths.^19–32^ In this fusion of insufficient knowledge, three themes were identified: normalization of pain as menstrual pain, incorrect diagnosis, and treatment was decided based on medical myths.

The women felt that the physicians dismissed their pain as a menstrual pain and as such something that was normal and that was part of being a woman, something all women live with. The women might receive the comment that they were unlucky that they belonged to the group of women who have severe menstrual pain. It also happened that the physicians believed that the women did not know what pain was (not experienced real pain) and therefore did not tolerate ordinary menstrual pain, even though the women had problems going to the toilet, having intercourse, *etc.*^19,20,23–25,27,29,31,32^

The normalization meant that physicians usually did not search for some other underlying cause of the pain. Instead, they often focused on the relief of symptoms. Sometimes lifestyle changes were recommended for example, physical exercise, and the physicians prescribed contraceptive pills to reduce bleeding and pain, even to very young teenage girls. Sometimes the physicians prescribed pain killers and sometimes the women themselves had to suggest the type of pain relief that suited them. Some women refused to take birth control pills because they wanted to know what caused their pain. By extension, the normalization led to the diagnosis being delayed.^18,20,22–26,28,30,31^

If the women's problem remained after treatment with painkillers and contraceptive pills, the physicians looked for simple causes. The physicians, however, were not inclined to accept that women's symptoms could be caused by any gynecological disorder or illness. Instead, the physicians gave other, incorrect diagnoses. The pain and the problem were located in the abdomen. Women who reported that they had simultaneous problems with the abdomen or intestines could receive diagnoses such as Irritable Bowel Syndrome, appendicitis, or muscle stretching. The problems could also be seen as mental. Some physicians thought the problem was in the head and not in the body. The women were diagnosed with depression or the physician blamed the symptoms on possible early abuse of alcohol or other drugs, on infection, or on previous miscarriage. Young women, students, were treated as being persons who did not take care of themselves.^19,23,26,29–32^

The women were referred to specialists such as psychiatrists or gastroenterologists before finally getting to meet a gynecologist. Some physicians put faith in so-called medical myths, which women knew were wrong, for example, that young women or teenage girls were too young to have endometriosis. Other myths concerned treatment when the women had been diagnosed. The women could receive the recommendation to remove the uterus, even though they were only in their twenties. Another myth that the physicians referred to was that the women's pains would be cured if they gave birth to children. Some arguments were that the hormones would restore the balance in the body or that the endometriosis would shrink and would not grow further.^20,22,24,26,27,29,31,32^

The recommendation for childbirth could be perceived as particularly offensive. Some women recalled that doctors recommended pregnancy as a treatment, even if they were infertile. Some other women were in such a situation that it was not suitable to have children at that time.

### Trivializing**—**just a women's issue

The women in these studies felt that no one took their problems seriously. Their discomfort was trivialized or completely disregarded. The physicians meant/felt that the symptoms were part of being a woman. The women's problems did not generate much interest and did not lead to continued investigation. The attitude was to take some painkillers and live as usual.

The approach of the physicians was described as offensive and sometimes blameful. There was little understanding of the fact that the problems could affect women's social life and quality of life.^21,23–27,29,31^

In this fusion of incorrect diagnoses three themes were identified: Not being taken seriously, no interest in women's problems, and insensitivity to women's situation.

The women felt that their symptoms were more difficult than painful menstruation and referred to their menstrual history and compared themselves with family and friends. The most common cause of dissatisfaction or anger by women was the feeling that physicians did not take their symptoms seriously. As their problems were not taken seriously, the physicians did not refer them for further investigation and treatment. The women felt denied since the physicians' attitude was that women exaggerated or imagined their symptoms, like having a form of “fantasy pain,” or had low pain thresholds. The women were requested to learn to handle their pain. Other women felt that the physicians wanted the women to feel that they had failed morally because they could not cope with their pains like other women. There was always a struggle to be taken seriously.^24–27,31,32^

The women who, after years of rejection and normalization of the pain, were referred for examination and received a diagnosis felt that they had finally received “evidence” that they had a disease and thus were able to discuss treatment options. The diagnosis was a relief. It was a confirmation that they had a disease and it was not just something in their head. However, even when the disease was verified with, for example, image evidence, physicians could question whether the pains really were proportional to the size of the lesions.^23–27,31,32^

The women felt that their symptoms were not very interesting or exciting for the physicians, or as it was expressed in a study**—**“endometriosis is not a sexy disease.” They felt invisible when the physicians were not interested in understanding them and their pain problems. Others felt that they were wasting their physicians' “time” because the physician believed that the trouble “was in the head”. The physicians showed lack of interest and distance when the women talked about their problems. They sighed, drummed their fingers on the table, avoided eye contact, and responded monotonously or with specialist terms. When simple explanations or jargon did not work, the physicians switched to normalizing or trivializing the problems.

The most difficult thing was when the physician moved the burden over to the women by asking how they wanted to be helped.^23–25,27,29–32^

There were also physicians who in a more brutal way showed their lack of interest. The physicians could argue that there was no diagnosis called menstrual pain, and that it was only stupid women who expressed themselves like this.^24–26,28,31,32^

The physicians could show insensitivity to the women's situation, which could be experienced as verbally, psychologically, and physical violations. For example, the physician could convey the information about the woman's endometriosis and infertility in an insensitive manner. It could be positive to get all this important information about endometriosis, but if the physician in the same breath mentioned infertility, it became too heavy. When the women raised the issue of children, they were abruptly informed that they would probably need to adopt. The physicians had not endeavored to find out about the women's life situation. There were women who had always longed to create a family but who were given the message about their infertility in an insensitive way, and this affected them for a long time to come. Some women even considered suicide because they had always wanted to have a family, and this dream had been totally crushed.^22–25,30–32^

The women described that they were in a difficult and vulnerable situation, filled with anxiety and frustration, which was mentally devastating. It was then difficult to be called stupid, crazy, and mentally ill.^22,24,25,27,28,31,32^

The women also felt physically vulnerable. Painful gynecological examinations could feel like abuse even though they were routine. The women did not want to undergo such examinations, but they had to.

### Competence promotes health

The third fusion describes positive experiences of meetings with physicians, and four studies contributed.^22,23,27,31,32^ Although the women mostly experienced ignorance and lack of interest in primary care, there were physicians who were interested in their problems. The insufficiency of knowledge became a minor concern if the women had a supportive relationship with their physician. Such physicians could help by listening and not delaying the referral to a specialist. One woman also said that the physician was not aware of endometriosis during the first visit but had done his homework before the next meeting.

Transferring from general to specialized care could offer a positive change. The women were reassured by knowing that they would be treated by physicians with special skills and competency. After several years of suffering they received a diagnosis and an explanation of why they had these problems. They acknowledged that the gynecologists showed that they cared, actively listened, and took them seriously. Such dedicated gynecologists took the time to explain and provide relaxation tips that could make the examination procedure easier. Women with fertility problems reported that their disease burden was alleviated by the physician communicating well about fertility. It also meant a lot that the gynecologist shifted focus from only the disease to how it affected the woman's life and situation. This strengthened and promoted women's health, helping them to continue their lives.

### Validation of the findings using a survey study

The analysts used a large qualitative survey study, including open questions to validate or “member check” the findings and interpretations from the metadata analysis/synthesis.^34^ This survey study included 135 women with endometriosis who evaluated a training program about social support, treatment, as well as professional and health care system performance. Because our study is a meta-analysis/synthesis of text, we did not have the opportunity to “member check” the findings with participants of the study. Since this survey study was performed with other participants and in another country (Germany),^35^ the findings from this survey study can be used for validation and triangulation of this meta-synthesis. The findings of this survey study pointed out some important factors.

Knowledge and competence were found to be among the most important factors when making the women with endometriosis feel confident and cared for. The second factor of importance was that the physicians/gynecologist believed in the women's descriptions about the symptoms and problems, that is, the women were taken seriously. The care encounters should be imbued with understanding and empathy. The third factor that was experienced as supportive was open and clear communication, based on interest and sensitivity. Obstacles and hindrances for women with endometriosis concerning managing their situation were ignorance, insufficient empathy, and nonsensitivity. These findings are in great agreement with those in the meta-synthesis, which strengthens its trustworthiness.

## Discussion

In this meta-synthesis, data from 14 research studies on women with endometriosis were reinterpreted to gain a deeper understanding of how they were perceived or about the health care encounter. We used the women's quotations presented as raw data and the text in the findings to draw an interpretation and gain an understanding of the phenomenon under study. It is interesting to note that even though the studies are from 2004 until 2018, the experiences of the women seem to remain. There are still the same issues and approaches perceived, but medical progresses are made.

The credibility (validity) of the data interpretations in this study is supported because they are the result of a systematic approach^13^ and can always be arbitrated for or against a variety of other interpretations. The data analyzed and interpreted in this study comprised primary research conducted on women from different cultures and contexts, but all having an endometriosis diagnosis. There are several other reviews that have synthesized qualitative research on how endometriosis affects women's lives and how they handle the problems.^10,36–39^ One of them focused on experiences of care and patient centering,^37^ whereas the others had a broader perspective. However, the selection of studies differs in part between our meta-synthesis and the other overviews. In addition, new studies have been added that are only included in our overview. The article by Dancet et al.^38^ also combined quantitative and qualitative articles, but in our meta-synthesis we only used qualitative articles. The fusions identified in our overview have not been highlighted as much in the other overviews. Some of our findings regarding experiences, how being approached from health care, are unique to women with endometriosis.

Insufficiency knowledge and its underlying themes could be found in the overview.^10^ This overview, however, emphasized mainly that the women sought knowledge and that the doctors compensated for their lack of knowledge by being more responsive. Insufficient knowledge was made visible in incorrect diagnoses and delayed diagnoses in three of the reviews,^9,36,37^ whereas the fourth found that it was about the lack of information.

The findings in this meta-synthesis may seem discouraging; the treatment of the women revealed perceived with ignorance about endometriosis and they experienced that their problems were trivialized; it was just a “women's issue.” This fusion was a strong theme based on the women's statements in the various studies that we included. In the overview articles, this “woman issue” was described as normalization of the problems and damaged wellbeing^10,38^ and also a cause of delayed diagnostics due to normalization and trivialization of women's problems.^37^ Dancet et al.^38^ mentions what lies in the subthemes of this fusion, namely to respect women's problems and to let the women be trusted. Others seem to be shared with persons with other types of chronic pain, mainly musculoskeletal and where most studies capture experiences from primary care. One common experience is the sense of not being believed since the pain is not visible or measurable was, for example, captured in qualitative evidence syntheses about chronic pain conditions^40^ and focusing on pelvic pain.^41^ The hard work to get a medical diagnosis was also central. Focusing on these issues there are some suggestions for developing endometriosis and pain centers, by using patient navigators, and working multidisciplinary.^42^

Our third fusion that competence promotes health, which is included in this theme, is the only fusion that brings forth some positive aspects acknowledged in the analyzed studies. That competency promotes health is often mentioned in one or two sentences here and there in the overviews.^10,36^ The fact that our three fusions are not clearly found in the overview articles that we compare in this study may be because these (three out of four) are merely descriptive and reflect what we already know. The fourth review article used “interpretation,” but on a combined material; quantitative and qualitative articles. This causes a methodological difficulty to extract material narratively/interpretatively from quantitative results.

We performed a meta-synthesis to, through interpretation, lift research results/findings to the next level and not only repeat what we already knew with some certainty.^14^ The meta-synthesis used in this study was grounded in hermeneutic theories.^16,34^ In this way, we clarified the women's experience of the encounter in endometrial care. We have interpreted and synthesized fusions and therefore it seemed relevant to discuss the results against other reviews—bringing forth synthesized data. Trying to broaden the one perspective view.

When we validated our fusions with the survey study,^35^ there was a finding showing that there are possibilities to make a positive difference regarding these women, by showing empathy and adopting a professional and competent approach. In a quite recently published article^43^ it was shown that medical education needs to equip physicians with the skills to acknowledge and incorporate women's knowledge of their bodies within the medical encounters. It was also highlighted that the women should be acknowledged and treated as partners in their health.

## Conclusion

The present meta-synthesis demonstrates that the “journey of endometriosis care” takes its starting point in primary care and there is a struggle to convince health care professionals that the symptoms are not ordinary menstrual pain and bleeding. There was also a struggle in that discomfort was trivialized and seen as women's issue. Transferring from general to specialized care could offer positive changes. The women were reassured by knowing that they would be treated by physicians with special skills and competency.

## Clinical Implication

Living with endometriosis and how one is perceived in the health care encounters have a great impact on these women's lives. The results of the present study highlight the importance of providing support for women who have endometriosis, so they are able to manage their everyday lives. Health care professionals need to be aware of endometriosis as a disease and be more sensitive for individual pain pattern among women, which could facilitate manageability. ESHRE^44^ presents clinical practice guidelines regarding medical progression, but health care professionals need to make their own clinical decisions based on their competency, considering the circumstances, and collaborate with the women.

## Abbreviation Used

HRQoL: health-related quality of life
